# Langerhans cell histiocytosis of the maxillae in a child treated only with chemotherapy: a case report

**DOI:** 10.1186/s13256-017-1286-3

**Published:** 2017-05-09

**Authors:** Angela Pia Cazzolla, Giuseppe Troiano, Khrystyna Zhurakivska, Eugenio Maiorano, Gianfranco Favia, Maria Grazia Lacaita, Giuseppe Marzo, Franca Dicuonzo, Stefano Andresciani, Lorenzo Lo Muzio

**Affiliations:** 1 Department of Translational Medicine, University of Bari, Bari, Italy; 2 Department of Clinical and Experimental Medicine, University of Foggia, Foggia, Italy; 3 Department of Pathological Anatomy, University of Bari, Bari, Italy; 4 Department of Life, Health & Environmental Sciences, University of L’Aquila, L’Aquila, Italy; 5 Department of Neuroradiology, Policlinico of Bari, Bari, Italy; 6 Department of Neurosciences, Policlinico of Bari, Bari, Italy; 7 Clinica Odontoiatrica, Via Rovelli 50, 71122 Foggia, Italy

**Keywords:** Langerhans cell histiocytosis, Children, Chemotherapy treatment, Case report

## Abstract

**Background:**

Langerhans cell histiocytosis is a sporadic disease caused by an uncontrolled pathogenic clonal proliferation of dendritic cells that have Langerhans cell characteristics. New treatment protocols provided by the HISTSOC-LCH-III (NCT00276757) trial show an improvement in the survival of children with langerhans cell histiocytosis.

**Case presentation:**

We report a case of Langerhans cell histiocytosis, which presented as an osteolytic lesion of the left pre-maxillae enclosing the deciduous incisor and canine in a 7-month-old white Italian boy. He was treated with chemotherapy. He achieved complete remission after 7 months and after 24 months no signs of recurrence were observed.

**Conclusions:**

As a result of this treatment, anesthetic sequelae and loss of teeth were avoided; in addition, we prevented a loss of the vertical dimension of occlusion.

## Background

Langerhans cell histiocytosis (LCH) is a sporadic disease caused by an uncontrolled pathogenic clonal proliferation of dendritic cells (DCs) that have Langerhans cell (LC) characteristics [1]. LCH mainly affects individuals in childhood, but can also be seen in adults. In Western Europe the annual incidence is estimated to be two to ten cases per 1 million children for ages from 0 to 15 years, with an almost equal distribution in both sexes.

The prognosis is closely related to the form in which the disease presents. For forms that affect high-risk organs, such as the liver, spleen, and/or bone marrow, the mortality rate is estimated at around 35% in patients who do not respond to therapy in the first 6 weeks [2]. Fortunately, new treatment protocols led to an improvement in the survival of children with LCH affecting high-risk organs as shown by the data provided by the HISTSOC-LCH-III (NCT00276757) trial [3–5].

## Case presentation

A 7-month-old white Italian boy presented with a painful swelling of the left side of his upper lip of 5 months’ duration. An intraoral examination revealed the presence of a swelling involving the alveolar bone of his anterior maxillae with high mobility of the deciduous central incisor.

**Fig. 1:**
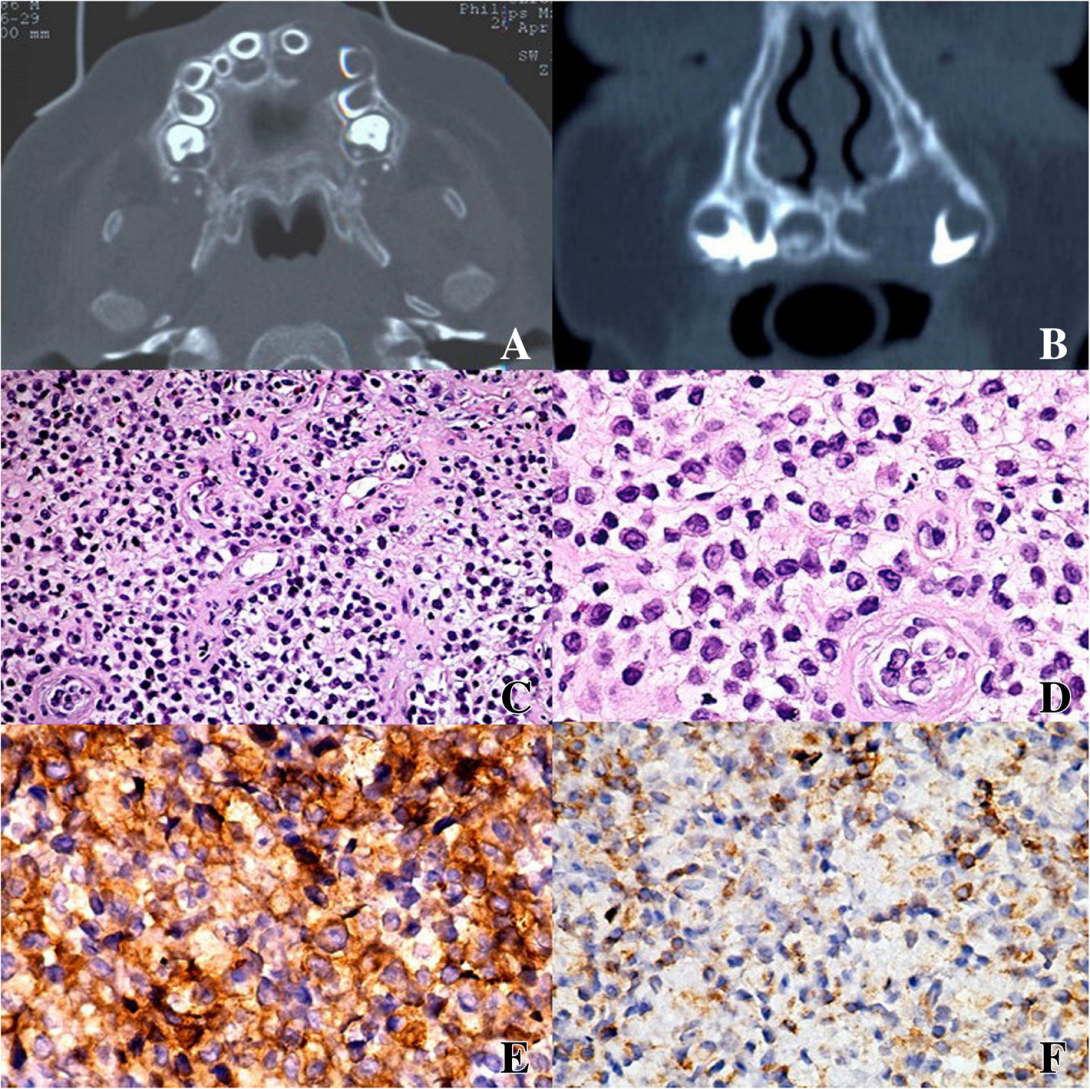
**a**, **b** Computed tomography scan showing a osteolytic bone lesion with poorly defined borders of the maxilla enclosing the deciduous incisor and canine, which resulted in the swelling of the alveolar cortical bone. **c**, **d** The lesion was composed of Langerhans cells with abundant cytoplasm and undefined cell borders, which were admixed with eosinophils and other inflammatory cells (**c** hematoxylin and eosin stain, original magnification ×10; **d** hematoxylin and eosin stain, original magnification ×20). **e** Immunohistochemical stain for Langerhans cell-specific CD1a antigen showing strong positive staining of neoplastic cells (original magnification ×20). **f** Mild positive staining for CD31 antigen (original magnification ×20)

Under general anesthesia, computed tomography (CT) and nuclear magnetic resonance imaging (NMRI) were performed, showing an osteolytic lesion of his left pre-maxillae enclosing the deciduous incisor and canine; the lesion did not present well-defined borders (Fig. 1a, b).

Under general anesthesia the upper incisor was removed and a biopsy of surrounding mucosa and intraosseous tissue was performed. Histopathologic examination revealed a diffuse infiltration of large pale-staining histiocytic cells interspaced with lymphocytes, plasma cells, and eosinophils (Fig. 1c, d). Immunohistochemical analysis showed positivity for CD1a, CD31, and S-100 antigens identifying histiocytes of Langerhans cells type (Fig. 1e, f). On the basis of these findings a final diagnosis of LCH was made.

Laboratory studies (complete blood cell count, hematocrit, hemoglobin, coagulation studies), liver function tests, urine osmolarity measurement after overnight water deprivation, chest radiography, and bone scintigraphy showed no evidence of other lesions, which excluded a multifocal and multisystem LCH.

Due to the extension of the lesion and the age of our patient, chemotherapy was chosen as treatment according to the current protocol (LCH-III) of the Histiocyte Society for patients with low-risk/multifocal bone disease or “special site” involvements. Treatment consists of two phases: a starting step for 6 weeks and a second step for 6 months. The starting step consisted of continuous prednisone (PDN) administered orally 10/m^2^ daily in three doses in a week, tapering over a period of 2 weeks and of vinblastine (VBL) 1.5 mg/m^2^ intravenous bolus on the first day of weeks 1, 2, 3, 4, 5, and 6. Subsequent therapy consisted of pulses of orally administered PDN 10 mg/m^2^ in three doses on days 1 to 5 every 3 weeks, starting at day 1 of week 7 until the end of month 6 from the start of therapy and VBL 1.5 mg/m^2^ intravenous bolus on day 1 every 3 weeks starting on day 1 of week 7 until the end of month 6 from the beginning of therapy. After such systemic therapy, some transitional collateral effects were observed. The most relevant were: mucositis, gastrointestinal toxicity immediately after the therapy, and a slight anemia that self-returned within a few months.

**Fig. 2:**
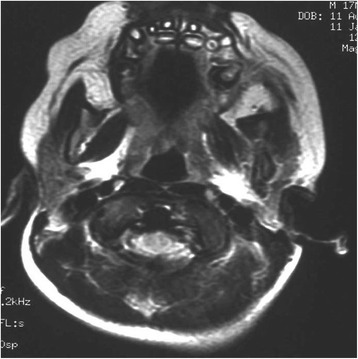
Magnetic resonance imaging performed after chemotherapy, showing no sign of soft tissue involvement with complete regression of the disease

**Fig. 3:**
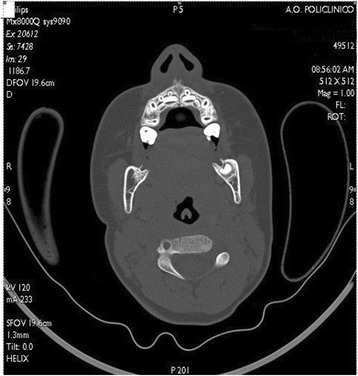
Computed tomography performed after chemotherapy, showing no sign of bone involvement with complete regression of the disease and a good position of the teeth involved in the neoplastic lesion

After initial treatment, cranial NMRI and CT scans revealed a reduction of bone lesion and after the continuation treatment an excellent response was achieved with complete remission after 7 months, as evident on NMRI (Fig. 2) and CT (Fig. 3) performed after therapy. In addition, no sign of recurrence has been found after 24 months of follow-up.

## Discussion

LCH is a proliferative disease of cells similar to LCs affecting individuals of any age, with extremely variable clinical manifestations [6, 7].

### Pathogenesis

The lesions are composed of cells with a dendritic LC phenotype [8].

Although many aspects of the etiology of LCH are still unknown, recent molecular studies have provided some clarification in understanding the pathogenic process. Knowledge about the nature of the disease was gained when 50 different pathologists observed that aberrant cells contained in the lesions have an appearance similar to histiocytes. Following these observations, Lichtenstein unified the various clinical pictures under the name of “Histiocytosis X” [6]. The true origin of these cells was better defined later due to the electron microscope. In 1982 Mireau *et al*. observed the presence of Birbeck granules in the histiocytes of LCH. These organelles would be responsible for processing and antigen presentation and appear to be exclusively present in LC [6]. This suggests a close link between the two cell types.

Another evidence that the abnormal cells are originated by LC came from the analysis of surface markers. For example, CD207, also known as Langerin [8], was identified; CD207 is normally expressed on the surface of LCs and associated with internalization of Birbeck granules [9]. In addition to CD207, the marker CD1a has been identified in pathological cells and normal LCs. For many years CD1a was considered specific to these cells and became the gold standard in the diagnosis of LCH [10, 11]. Subsequent research, however, denied its specificity in identifying LCs, as CD1a markers have also been identified in other cell subsets [12].

As known, the main function of LCs is to monitor the epidermis by the presence of foreign antigens [13, 14]. The presence of such antigens triggers the activation of a series of cells responsible for defense and the secretion of cytokines and other ligands recognized by immune cell receptors. Once activated, the LCs process the antigen and migrate to regional lymph nodes, where they present the antigen to T cells, thus activating the adaptive immunity chain. In the absence of external stimulation, LCs express on their surface the CCR6 receptor; this ligand is secreted by epidermal keratinocytes. After activation of LCs, this receptor undergoes a downregulation with the simultaneous upregulation of the CCR7 receptor that shows affinity for ligand CCL19 and CCL21 secreted by cells of the lymph nodes [6].

Analyzing the cells that infiltrate the various organs during LCH, two independent studies have confirmed that these abnormal cells have an altered expression of chemokine receptors CCR6 and/or CCR7 [15, 16].

Another alteration of the immune response in the course of LCH was found in the response of T lymphocytes, recording an expansion of regulatory T cells. This happens because the pathological DCs are not efficient in antigen presentation and have a low rate of proliferation [17, 18].

Despite the remarkable similarities to LCs, recent gene expression analyses of LCH cells showed that these abnormal cells are not derived from the LCs, but probably originate from myeloid DCs, which express the same antigens (CD1a and CD207) of the skin LC [19, 20].

The close correlation with the cells of the immune system has initially directed research toward the immune and inflammatory origin of LCH. Even today this remains the most important issue, that is: Is the clonal proliferation of LCH cells a result of malignant transformation or is it a result of an immunological stimulus?

In support of the hypothesis that LCH is a clonal neoplastic disorder, recent discoveries have shown the V600E mutation in the *BRAF* oncogene in LCH cells, the same mutation found in other tumor types [21]. In addition, almost all lesions show evidence of activated ERK downstream of *BRAF*. In all the lesions, it was found that the extracellular signal-related (ERK) pathway is activated, including cases *BRAF* V600E-negative. This leads one to suspect that there are other mutations of the chain Ras-Raf-MEK-ERK pathway [22, 23].

These genetic findings may have an important clinical implication. For example, they might clarify the diagnosis, discriminating high-risk versus low-risk disease [24], and define the clinical course of the disease; they would allow a more targeted therapy, such as BRAF inhibitors (for example, vemurafenib and dabrafenib), or the combination of BRAF inhibitors plus MEK inhibitors. Despite promising results, further experiments are required before the therapies can be applicable to adults and children [25–27].

### Clinical manifestation

LCH disease can affect different organs and systems, resulting in highly variable symptoms and signs. In Western Europe the annual incidence is estimated to be two to ten cases per 1 million children for ages from 0 to 15 years, with an almost equal distribution in both sexes. The clinical picture ranges from the most benign when there is only bone involvement, with single or multiple osteolytic lesions, to forms that are very debilitating, like Hand–Schüller–Christian disease, which is characterized by the triad of bone lesions, exophthalmos, and polyuria, or the fulminant disease called Letterer–Siwe disease, which impairs the functioning of internal organs and presents with hepatosplenomegaly, lymphadenopathy, bone lesions, skin rash, and pancytopenia [28].

However, from a practical point of view that is useful for treatment and prognosis, a primary distinction must be made between the following two forms: single-system LCH, involving a single organ in a single or multiple sites and multisystem LCH that affects multiple organs or systems, including bone, abdominal/gastrointestinal system (liver and spleen), lungs, bone marrow, endocrine system, eyes, central nervous system (CNS), skin, and lymph nodes. In addition, the organs involved can be divided into those at high risk, such as liver, spleen, and bone marrow, and those at low risk, which include skin, bone, lungs, lymph nodes, gastrointestinal tract, pituitary gland, and CNS [2].

The single-system form affects approximately two-thirds of pediatric patients with LCH and usually involves bones, skin or, more rarely, lymph nodes. Single bone lesions conventionally are treated with a surgical curettage associated or not with local instillation of corticosteroids. This treatment is, in many cases, resolutive and relieves the clinician from having to resort to more invasive treatments, commonly used in the past, which can cause further complications. If lesions occur in the craniofacial bones, defined as sites with high risk of nervous system involvement, or bone sites that are difficult to access or when there is a high risk of fracture, as well as in cases of multiple bone lesions or a single but very massive lesion, systemic chemotherapy is indicated [6, 29]. The use of systemic chemotherapy also becomes necessary in cases of multisystem involvement.

### Oral manifestations of LCH

The oral cavity may sometimes be the first or the only site of LCH manifestation. Here it can present with ulceration of the oral mucosa, which is associated with lymphadenopathy, periodontal defects, dental hypermobility, or premature loss of teeth [30]. Maxilla and mandible, along with the other bones of the skull, are the most frequently affected bone sites. Intraosseous lesions are found mainly in the body and mandibular branch and may be symptomatic or not. The mucosa may present as erythematous, inflamed, or ulcerated. Cervical lymphadenopathies are encountered in 30% of patients with oral lesions [31].

The diagnosis is made on histological report, supported by clinical and radiographic examination [30]. At immunohistochemical analysis, the histiocytic cells show positivity for S-100 markers and/or CD1a, and show ATPase activity of the cellular membrane [32, 33]. There are no specific laboratory tests for the diagnosis of LCH. However, blood tests (such as complete blood count and platelet count), liver function tests, and urine analysis can be useful to estimate the extent and severity of the disease. Imaging studies that can be useful are: conventional X-ray, CT, and magnetic resonance imaging (MRI) of the affected areas. A CT scan, in particular, is indicated when there is suspected involvement of the skull bones [2, 30, 34].

A biopsy is needed to diagnose LCH. It is frequently done on bone lesions, epidermal sites, and lymph node sites. A liver biopsy may be indicated if blood analyses reveal hypoalbuminemia without other apparent cause, elevated bilirubin, or elevated liver enzymes [2]. At histological examination LCH cells appear as large round or oval mononuclear cells with a vesicular nucleus and have a moderate amount of eosinophilic plasma. Other cells present in the lesions are: lymphocytes, mononuclear phagocytes, and abundant eosinophils [30].

### Treatment

The prognosis is closely related to the form in which the disease presents. For forms that affect high-risk organs, such as liver, spleen, and/or bone marrow, the mortality rate is estimated at around 35% in patients who do not respond to therapy in the first 6 weeks [2]. Fortunately, new treatment protocols led to an improvement in the survival of children with LCH affecting high-risk organs as shown by the data provided by the HISTSOC-LCH-III (NCT00276757) trial [3–5].

The choice of treatment, topical or systemic, takes into account the site and extent of the disease. Guide protocols for the treatment of patients with LCH are defined by international multicenter clinical studies, LCH I-II-III, and are being developed by the LCH-IV trial.

Unifocal bone lesions are the predominant clinical form of LCH. The choice of approach should take account of the symptoms, the organ affected, and the size of the lesion [35]. For single bone lesions, curettage alone or curettage associated with injections of methylprednisolone can be decisive [2]. This applies for small lesions (<2 cm). For large lesions, however, surgical excision is not indicated, as it increases the risk of permanent bone defects with prolonged healing times. The involvement of a critical anatomical site, like skull bones, may justify systemic therapy. The most commonly used systemic approach consists of steroids and VBL, a combination relatively non-toxic and well tolerated [35].

According to the LCH-III trial, high-risk patients should be subjected to 12 months of chemotherapy, while those at low risk with lesions at critical sites, such as in the mandible, where an extensive surgery could destroy any possibility of secondary development of the teeth, 6 months of systemic therapy with VBL and PDN is recommended to limit the risk of sequelae and recurrences [2].

The evaluation of response to therapy makes use of clinical observations, such as the absence of pain and other symptoms, as well as radiographic examinations, which are often difficult to interpret. Bone lesions, in particular, can take many months before there are radiographic signs of healing; a good sign is the appearance of sclerosis in the periphery of the lesion [2].

Although LCH is a relatively benign disease, the affected organs may have residual sequelae. Children with a history of LCH should be monitored until adulthood. In particular, if the disease was localized in the jaw bones, it is recommended that the development of teeth and bones be followed even after healing. Regarding the follow-up, all patients should be followed for 5 years after the end of therapy or until their growth and pubertal development is complete [35].

In the reported case, the patient showed a full response at the end of the first 6 weeks of treatment confirming the strength of LCH-III protocols. According to the Histiocyte Society guidelines, we suggest multi-agent chemotherapy for extensive involvement of the jaws to avoid “heroic surgery”, loss of teeth, anesthetic sequelae, and the loss of the vertical dimension of occlusion. In addition, the minimal necessary follow-up should be made at least every month in the first year after total disease remission and subsequently 6 monthly for 2 to 5 years before considering the patient to be entirely free of disease.

## Conclusions

In our case report of LCH in a 7-month-old boy, chemotherapy was the best treatment; he had a complete remission and because of the pharmacologic therapy an excellent response was achieved. In this way we avoided a lot of important sequelae such as loss of teeth, loss of vertical dimension of occlusion, and we also avoided surgery so that the young patient had less discomfort.

## Abbreviations

CNS: Central nervous system
CT: Computed tomography
DC: Dendritic cell
LC: Langerhans cell
LCH: Langerhans cell histiocytosis
MRI: Magnetic resonance imaging
NMRI: Nuclear magnetic resonance imaging
PDN: Prednisone
VBL: Vinblastine
